# Phaeochromocytoma in a teenage girl with Cushing’s Syndrome

**DOI:** 10.1186/1687-9856-2013-S1-P138

**Published:** 2013-10-03

**Authors:** Suryono Yudha Patria, P Bagaswoto, Suripto

**Affiliations:** 1 Div. Pediatric Endocrinology, Dept. Child Health; Fac. Medicine, Gadjah Mada University, Yogyakarta, Indonesia; 2 Dept. Radiology and Radiotherapy; Fac. Medicine, Gadjah Mada University, Yogyakarta, Indonesia; 3 Dept. Pathology, Fac. Medicine, Gadjah Mada University, Yogyakarta, Indonesia

## 

External excess of corticosteroid is frequently the cause of Cushing’s syndrome, while less report of such case due to internal tumor of adrenal gland. Here, we report clinical presentation, and etiological diagnosis of a 14 year old girl with Cushing’s syndrome caused by supra renal tumor.

The first clinical presentation were recurrent cephalgia, irregular periods, hirsutism, skin striae, moon-face, acne, hypertension (140/90 mmHg), and overweight. All symptoms and signs have suggested an excess of corticosteroid hormone, and those occured for almost for one year. Laboratory examination showed normal of random blood glucose level (113 g/dL), and electrolytes (Na: 140,4 mmol/L, K: 3,7 mmol/L, Cl: 107,8 mmol/L). Morning corticosteroid hormone analysis was found increasing (32.0 ug/dL, normal range 2.5 – 25), and dexamethasone test failed to suppress hypothalamic-pituitary-suprarenal axis (pre-test cortisol 31.9 ug/dL, and post-test: cortisol 32.1 ug/dL). All laboratory findings suggested an internal corticosteroid excess. The next, ultrasound image showed a profound mass at left suprarenal gland, and normal of both kidneys. Futher, opened biopsy of the tumor showed macroscopically very fragile mass and tend to hemorrhage. Pathological study concluded a phaeochromocytoma in left supra renal gland. These results suggest that phaeochromocytoma was the cause of corticosteroid excess and presented clinically as Cushing’s syndrome in our case.

